# Adverse cardiovascular events associated with biodegradable polymer drug-eluting stents and durable polymer everolimus-eluting stents: A systematic review and meta-analysis of 10 randomized controlled trials

**DOI:** 10.1097/MD.0000000000007510

**Published:** 2017-07-14

**Authors:** Pravesh Kumar Bundhun, Girish Janoo, Chandra Mouli Yanamala, Feng Huang

**Affiliations:** a Institute of Cardiovascular Diseases, The First Affiliated Hospital of Guangxi Medical University; b Guangxi Medical University, Nanning, Guangxi, P.R. China; c Department of Internal Medicine, Ealing Hospital, University of Buckingham, Southall, London, United Kingdom.

**Keywords:** biodegradable polymer drug-eluting stents, cardiovascular events, coronary artery diseases, durable polymer everolimus-eluting stents, percutaneous coronary intervention, randomized controlled trials, stent thrombosis

## Abstract

**Background:**

Controversies have been observed among network meta-analyses comparing biodegradable polymer drug-eluting stents (BP-DES) with durable polymer drug-eluting stents (DP-DES). We aimed to compare the adverse cardiovascular events associated with BP-DES and durable polymer everolimus-eluting stents (DP-EES) using a large number of patients obtained from randomized controlled trials (RCTs).

**Methods:**

Electronic databases were searched for randomized trials comparing BP-DES with DP-EES. Adverse cardiovascular outcomes observed between 6 months and 3 years were considered as the clinical endpoints in this analysis. Odds ratios (ORs) with 95% confidence intervals (CIs) were calculated and the pooled analyses were performed with RevMan 5.3 software. All authors had full access to the data, and they have read and agreed to the manuscript as written.

**Results:**

Ten trials involving a total number of 13,218 patients (7451 patients treated by BP-DES and 5767 patients treated by DP-EES) were included. No significant difference was observed when analyzing mortality and myocardial infarction between BP-DES and DP-EES with OR 1.08, 95% CI 0.87–1.34, *P* = .47 and OR 1.04, 95% CI 0.84–1.28, *P* = .72 respectively. Target vessel revascularization, target lesion revascularization, major adverse cardiac events, and stroke were also not significantly different with OR 1.11, 95% CI 0.92–1.33, *P* = .28; OR 1.11, 95% CI 0.94–1.33, *P* = .22; OR 1.12, 95% CI 0.99–1.27; *P* = .07; and OR 1.13, 95% CI 0.69–1.84; *P* = .62 respectively. In addition, total stent thrombosis (ST) was similarly reported between BP-DES and DP-EES with OR 0.85, 95% CI 0.59–1.21; *P* = .37. However, even if BP-DES were associated with a higher rate of definite ST with OR 1.69, 95% CI 0.92–3.08, *P* = .09 and DP-EES were associated with a higher rate of probable ST with OR 0.67, 95% CI 0.38–1.17, *P* = .16, these results were not statistically significant.

**Conclusions:**

Between 6 months and 3 years, BP-DES were similar in terms of cardiovascular outcomes compared to DP-EES. However, further long-term follow-up research is recommended.

## 1 Introduction

Controversies have been observed among network meta-analyses comparing biodegradable polymer drug-eluting stents (BP-DES) with durable polymer drug-eluting stents (DP-DES). To be more precise, the Bayesian approach network meta-analysis comparing BP-DES with bare metal stents (BMS) and DP-DES, respectively, in patients undergoing coronary revascularization showed durable polymer everolimus-eluting stents (DP-EES) to be safer than biodegradable polymer biolimus-eluting stents (BP-BES) at 1-year follow-up.^[1]^ BP-BES were associated with a higher risk of stent thrombosis (ST) compared to DP-EES. Another example is the comprehensive network meta-analysis, which aimed to investigate the efficacy and safety of BP-BES with DP-DES using data from 60 randomized controlled trials (RCTs), which showed that even if BP-BES and DP-EES were equally effective, DP-EES were considered safer than BP-BES.^[2]^ In addition, the mixed treatment comparison meta-analysis comparing BP-DES with DP-DES showed DP-EES to be the most effective and safest DES^[3]^ compared to the other DES analyzed. However, the authors also concluded that the utility of BP-DES in the context of excellent adverse clinical outcomes with newer-generation DP-DES for example DP-EES needed to be further confirmed in future studies. Hence, we aimed to compare the adverse cardiovascular events associated with the implantation of BP-DES and DP-EES during a mean follow-up period ranging from 6 months to 3 years, using a large number of patients obtained from randomized trials.

## 2 Methods

### 2.1 Data sources and search strategy

The Cochrane Library, PubMed/Medline, and EMBASE databases were searched for trials comparing BP-DES with DP-EES by typing terms such as “Biodegradable and durable drug eluting stents”. Abbreviations such as “DES and EES” were also used. Moreover, the words “durable DES” were also replaced by the words “permanent DES” and another search was carried out. In addition, reference lists of suitable studies were also checked for relevant trials. To ensure a better search, official websites of several well-known journals related to Cardiology such as the Journal of the American College of Cardiology and Circulation were also searched for any new or missing trial. Only articles published in English were considered and this search process was terminated by the end of March 2016.

### 2.2 Inclusion and exclusion criteria

Studies were included if:

(a) They were RCTs comparing BP-DES with DP-EES.
(b) They reported adverse cardiovascular outcomes as their clinical endpoints.
(c) They had a follow-up period of ≥ 6 months.

Studies were excluded if:

(a) They were non-RCTs (observational studies, meta-analyses, case studies, letter to editors).
(b) They did not compare BP-DES with DP-EES.
(c) They did not report adverse cardiovascular outcomes.
(d) They had a follow-up period of < 6 months.
(e) They were duplicates or they were associated with the same trial.

### 2.3 Outcomes, definitions, and follow-up

Adverse cardiovascular outcomes were considered as the clinical endpoints in this meta-analysis. They included:

(a) All-cause mortality (cardiac and noncardiac death)
(b) Myocardial infarction (MI)
(c) Target vessel revascularization (TVR)
(d) Target lesion revascularization (TLR)
(e) Stroke
(f) Major adverse cardiac events (MACEs) consisting of death, MI, and revascularization (TVR and TLR).
(g) ST which was defined according to the Academic Research Consortium (ARC)^[4]^ and involved definite ST, probable ST, and total ST (definite and probable).

This analysis had a mean follow-up period ranging from 6 months to 3 years. One trial had a follow-up period of 6 months, 2 years, and 3 years, respectively, whereas 7 trials had a follow-up period of 1 year (Table 1).

**Table 1:**
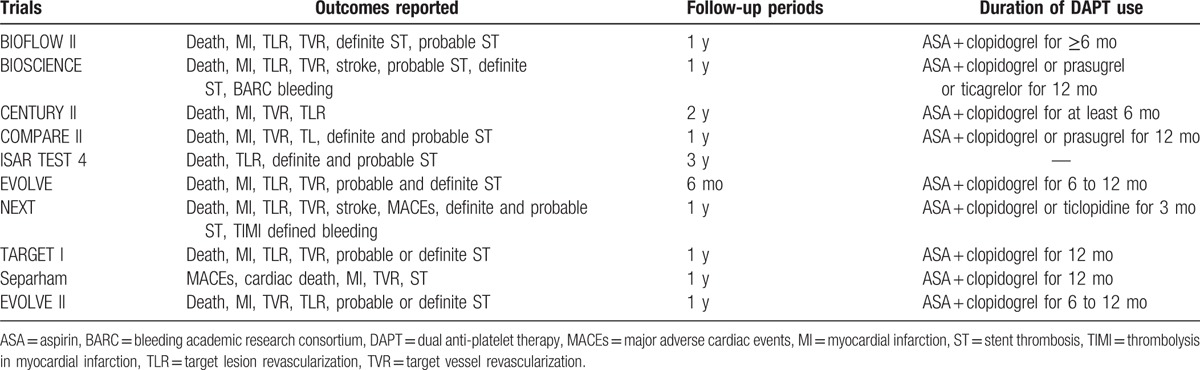
Reported outcomes and follow-up periods.

### 2.4 Data extraction and quality assessment

Three authors (PKB, GJ, and CMY) independently reviewed and assessed the methodological quality of each trial, which was considered eligible for this systematic review and meta-analysis. Information and data concerning the trial name, trial unique identifier number, total number of patients randomized to BD DES and DP-EES, respectively, patients’ enrollment periods, data concerning the baseline characteristics of the patients included, the clinical endpoints reported as well as the follow-up periods of each eligible trial were carefully extracted. Disagreements were solved by the third author (FH). The bias risk was assessed by the authors in accordance to the recommendations by the Cochrane Collaboration^[5]^ and grades were allocated accordingly to these trials. Trials were allocated a grade “A” if a very low risk of bias was reported, a grade “B” if a low risk of bias was noted, a grade “C” if a moderate risk of bias was observed, and a grade “D” if a high risk of bias was noted. The authors tried to be fair enough during this assessment/grading process. Bias grades have been listed in Table 2.

**Table 2:**
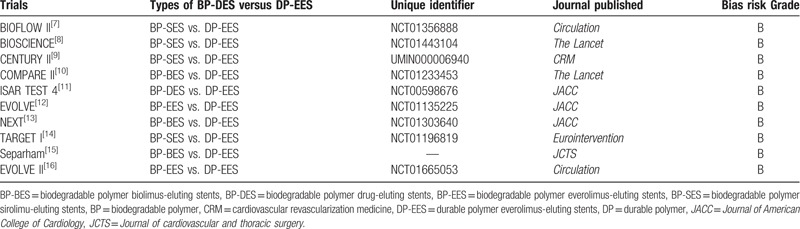
General features of the trials included in this study (part 1).

### 2.5 Methodological and statistical analysis

The Preferred Reporting Items for Systematic Reviews and Meta-Analyses (PRISMA)^[6]^ guideline was followed for this systematic review and meta-analysis of randomized trials. Heterogeneity among the subgroups analyzing adverse cardiovascular events was assessed using the Cochrane Q-statistic and the *I*^2^ statistic tests. In this analysis, a *P* value ≤.05 was considered statistically significant, whereas a *P* value >.05 was considered statistically insignificant. In addition, a very low heterogeneity was indicated by an *I*^2^ value of 0%, whereas larger values of *I*^2^ indicated increased heterogeneity. A fixed or random effect model was used depending on the value of *I*^2^ obtained. Odds ratios (ORs) with 95% confidence intervals (CIs) were calculated appropriately and the analyses were conducted with RevMan 5.3 software. All authors had full access to the data included in this analysis, and they have read and agreed to the manuscript as written.

Sensitivity analysis was conducted by excluding these trials one by one and performing another analysis to observe any significant changes in the results obtained.

### 2.6 Publication bias assessment

Funnel plots obtained from Revman were used to visually observe any publication bias. As this analysis involved only 10 trials (which was considered a smaller volume of trials), funnel plots were considered relevant enough to assess publication bias.

### 2.7 Ethics approval and patients consent

Ethical approval and patient consents were not necessary for systematic reviews and meta-analyses.

## 3 Results

### 3.1 Search results

**Figure 1:**
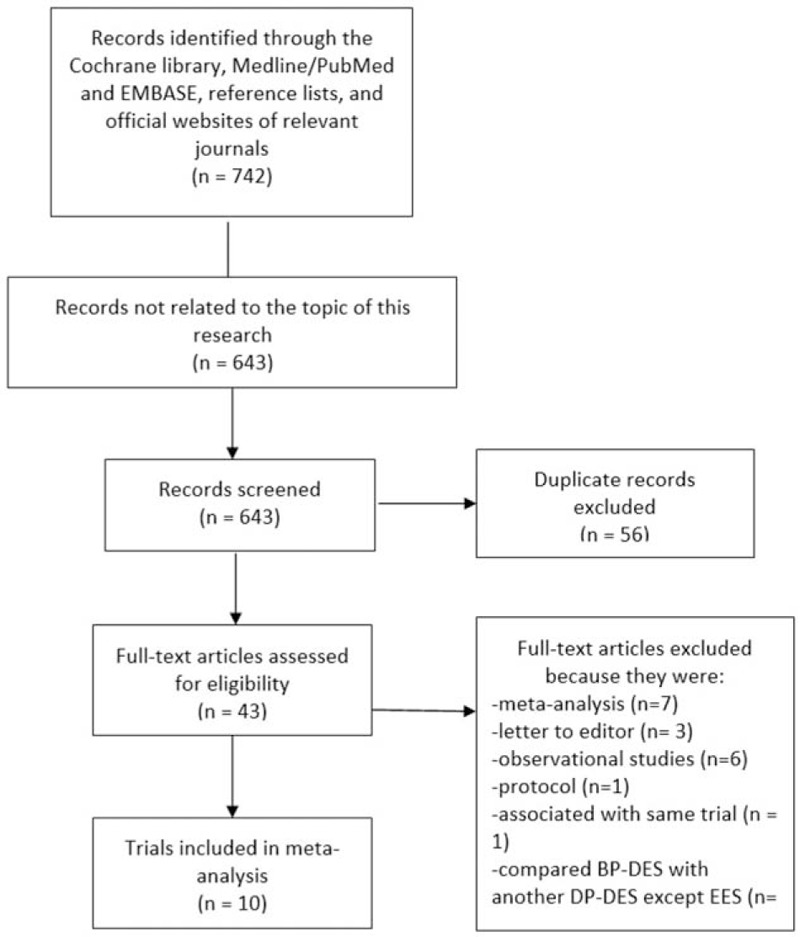
Flow diagram showing the process of study selection.

A total number of 742 articles were obtained from the Cochrane Library, PubMed/Medline, and EMBASE databases, as well as from the reference lists of suitable articles and from official websites of well-known cardiology journals. After a careful assessment of the titles and abstracts, 699 articles were eliminated as they were either not related to the topic of this research or they were duplicates. A total of 43 full-text articles were assessed for eligibility. After reviewing the full-text articles, further articles were eliminated since: 7 articles were meta-analyses, 3 articles were letter to editors, and 6 articles were observational studies. In addition, 15 more articles were eliminated as they compared BP-DES with either durable polymer sirolimus-eluting stents, or durable polymer paclitaxel-eluting stents. Another 2 articles were eliminated as one was a design of a trial, whereas the other was associated with the same trial. Finally, 10 trials^[7–16]^ were selected for this analysis. This study selection process has been represented in Fig. 1.

### 3.2 General features of the trials included

A total number of 13,218 patients (7451 patients treated by BP-DES and 5767 patients treated by DP-EES) were included. The types of BP-DES involved, the unique identifier number as well as the journal in which these trials were published have been listed in Table 2, whereas Table 3 summarized the patients’ enrollment periods, and listed the total number of patients treated with BP-DES and DP-EES, respectively.

**Table 3:**
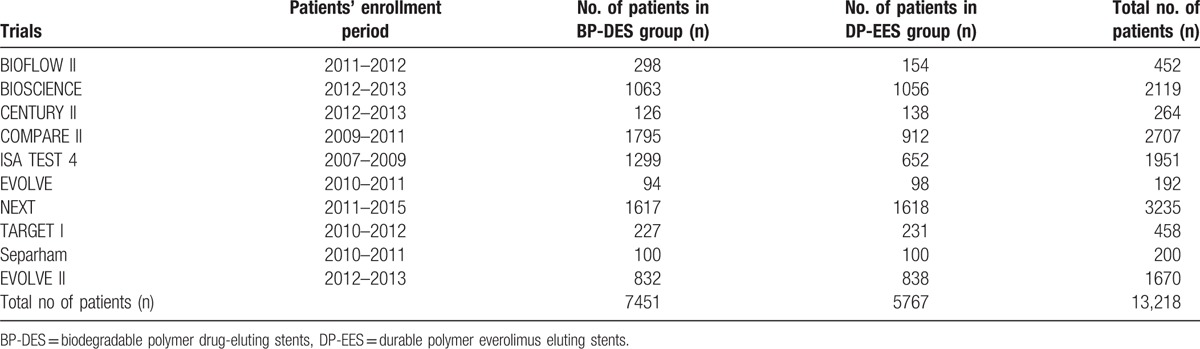
General features of the trials included in this study (part 2).

### 3.3 Baseline features of the trials included

The baseline characteristics of the patients have been summarized in Table 4.

**Table 4:**
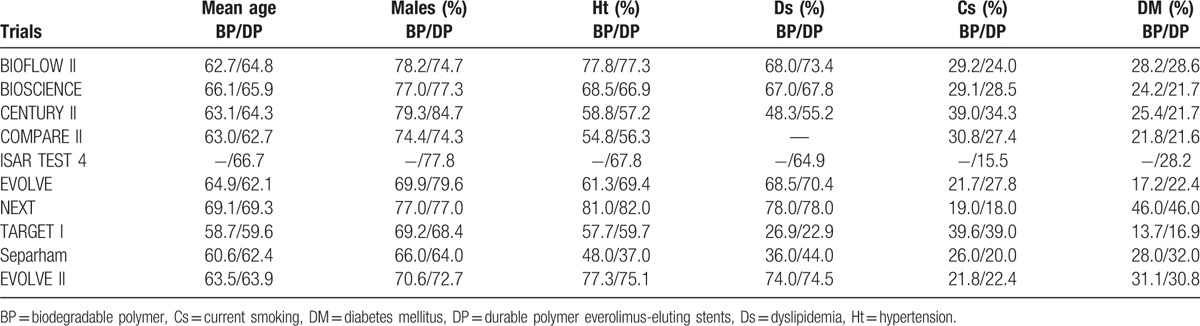
Baseline features of the trials included in this analysis.

Trial CENTURY II consisted of the majority of patients who were males. Trial NEXT had the highest number of patients with hypertension and diabetes mellitus, respectively. The percentage of patients with dyslipidemia varied considerably among the different trials. For example, NEXT trial showed a high percentage of patients with dyslipidemia in both groups, whereas TARGET I trial showed a very low percentage of patients with dyslipidemia, which could have been because of early treatment with statin or a decrease in the level of high-density lipoprotein. According to Table 4, there were no significant differences in baseline features among patients randomized to either the BP-DES or DP-EES group.

### 3.4 Comparing the adverse cardiovascular events associated with BP-DES and DP-EES

**Figure 2:**
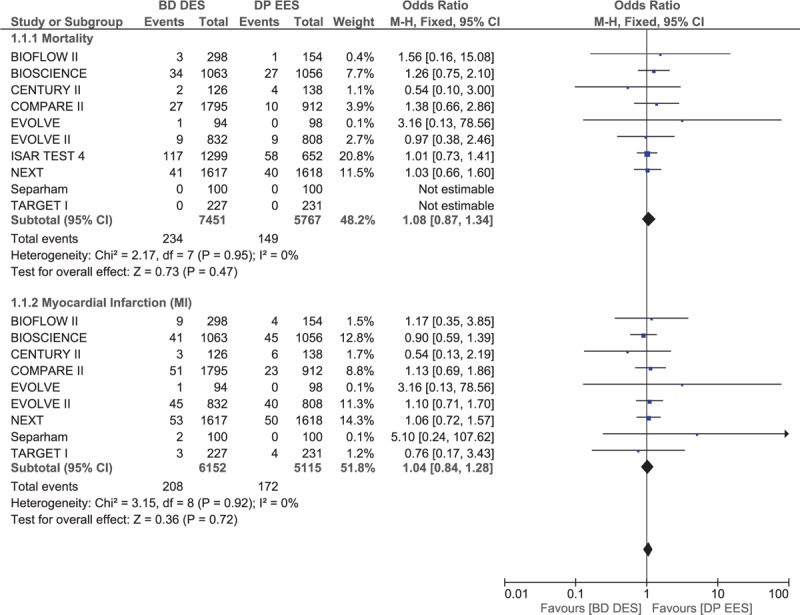
Comparing mortality and myocardial infarction between biodegradable polymer drug-eluting stents (BP-DES) and durable polymer everolimus-eluting stents (DP-EES).

Results of this analysis showed that no significant difference in mortality and MI between BP-DES and DP-EES with OR 1.08, 95% CI 0.87–1.34, *P* = .47, *I*^2^ = 0% and OR 1.04, 95% CI 0.84–1.28, *P* = .72, *I*^2^ = 0%, respectively. This result has been illustrated in Fig. 2.

**Figure 3:**
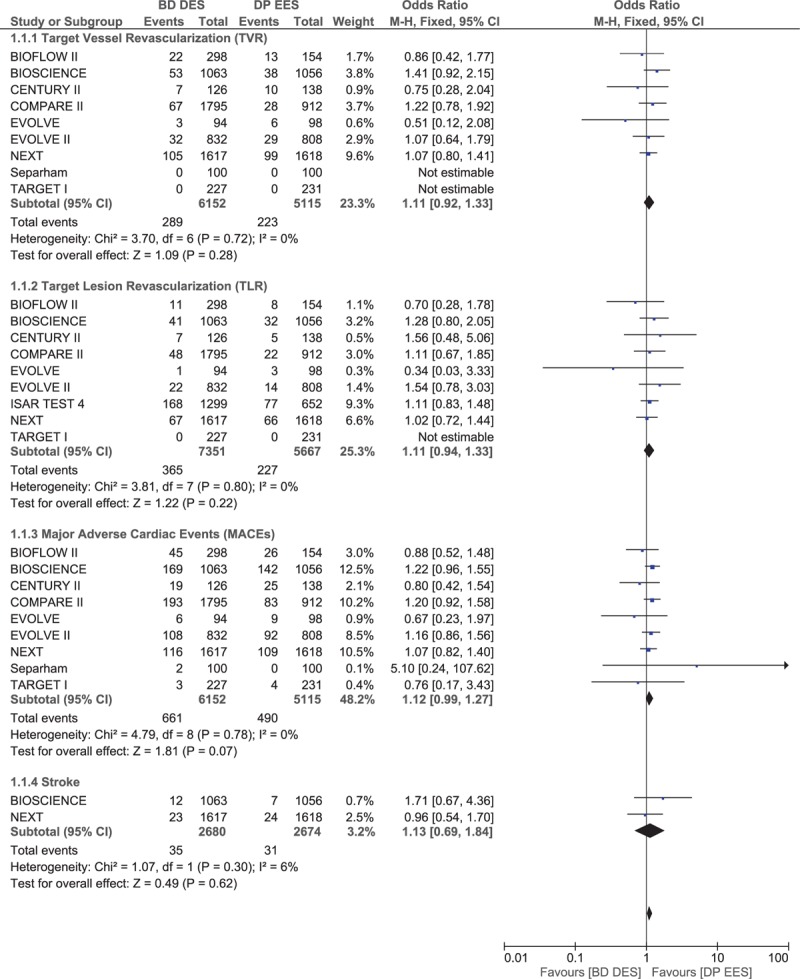
Comparing the other adverse cardiovascular events between biodegradable polymer drug-eluting stents (BP-DES) and durable polymer everolimus-eluting stents (DP-EES).

TVR, TLR, MACEs, and stroke were also not significantly different with BP-DES and DP-EES, with OR 1.11, 95% CI 0.92–1.33, *P* = .28, *I*^2^ = 0%; OR 1.11, 95% CI 0.94–1.33, *P* = .22, *I*^2^ = 0%; OR 1.12, 95% CI 0.99–1.27, *P* = .07, *I*^2^ = 0%; and OR 1.13, 95% CI 0.69–1.84, *P* = .62, *I*^2^ = 6%, respectively. These results have been represented in Fig. 3.

### 3.5 Comparing ST associated with BP-DES versus DP-EES

**Figure 4:**
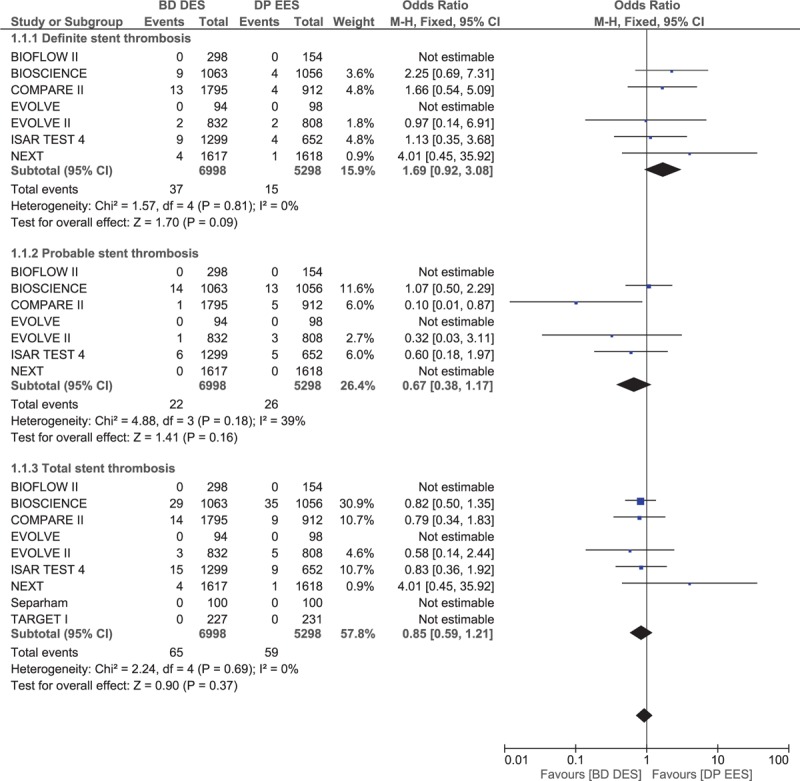
Comparing stent thrombosis between biodegradable polymer drug-eluting stents (BP-DES) and durable polymer everolimus-eluting stents (DP-EES).

Total ST (definite + probable) was not significantly different between BP-DES and DP-EES with OR 0.85, 95% CI 0.59–1.21, *P* = .37, *I*^2^ = 0%. BP-DES were associated with a higher rate of definite ST with OR 1.69, 95% CI 0.92–3.08, *P* = .09, *I*^2^ = 0%. However, probable ST was higher in the DP-EES group with OR 0.67, 95% CI 0.38–1.17, *P* = .16, *I*^2^ = 39%. However, in both cases, the results were not statistically significant. These results have been represented in Fig. 4.

### 3.6 Comparing BP-SES with DP-EES

**Figure 5:**
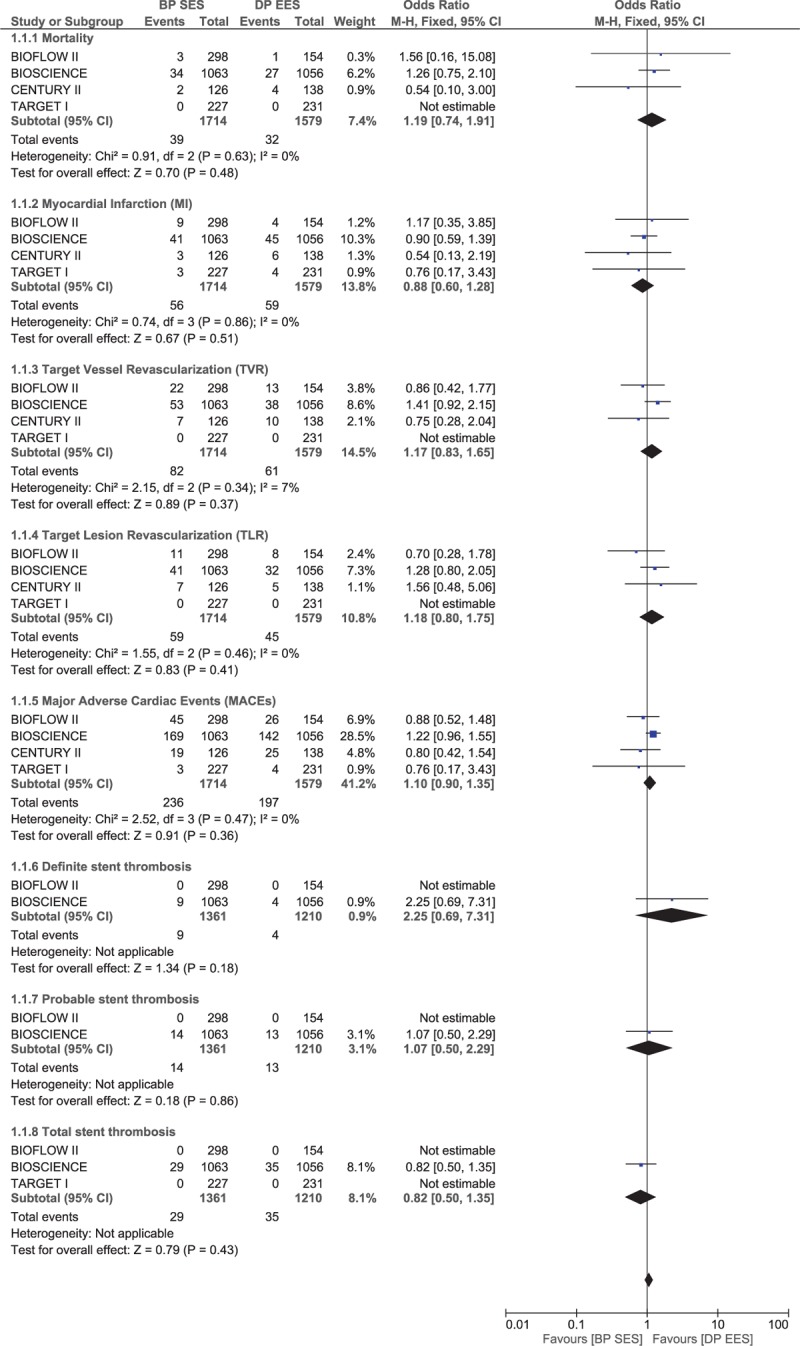
Comparing the adverse cardiovascular events between biodegradable polymer drug-eluting stents (BP-SES) and durable polymer everolimus-eluting stents (DP-EES).

This further analysis comparing BP-SES with DP-EES also did not show any significant difference between BP-SES and DP-EES among all the clinical outcomes analyzed. Mortality, MI, TVR, TLR, and MACEs were not significantly different with OR 1.19, 95% CI 0.74–1.91, *P* = .48, *I*^2^ = 0%; OR 0.88, 95% CI 0.60–1.28, *P* = .51, *I*^2^ = 0%; OR 1.17, 95% CI 0.83–1.65, *P* = .37, *I*^2^ = 7%; OR 1.18, 95% CI 0.80–1.95, *P* = .41, *I*^2^ = 0%; and OR 1.10, 95% CI 0.90–1.35, *P* = .36, *I*^2^ = 0%, respectively. ST was also not significantly different between these 2 types of stents. Results comparing BP-SES with DP-EES have been represented in Fig. 5.

### 3.7 Comparing BP-BES with DP-EES

**Figure 6:**
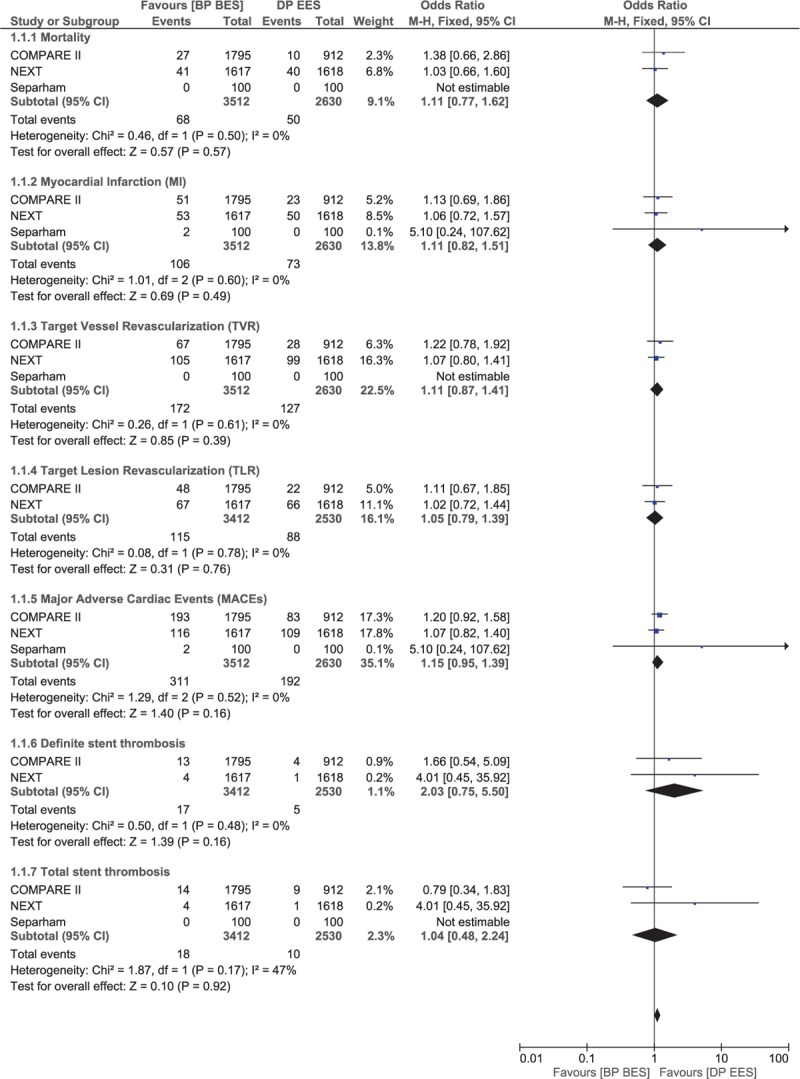
Comparing the adverse cardiovascular events between biodegradable polymer drug-eluting stents (BP-BES) and durable polymer everolimus-eluting stents (DP-EES).

When BP-BES were separately compared with DP-EES, no significant differences were observed in mortality, MI, TVR, TLR, and MACEs with OR 1.11, 95% CI 0.77–1.62, *P* = .57, *I*^2^ = 0%; OR 1.11, 95% CI 0.82–1.51, *P* = .49, *I*^2^ = 0%; OR 1.11, 95% CI 0.87–1.41, *P* = .39, *I*^2^ = 0%; OR 1.05, 95% CI 0.79–1.39, *P* = .76, *I*^2^ = 0%; and OR 1.15, 95% CI 0.95– 1.39, *P* = .16, *I*^2^ = 0%, respectively. Even the results for ST were not significantly different. Results comparing BP-BES with DP-EES have been represented in Fig. 6.

Results of this analysis have been listed in Table 5.

**Table 5:**
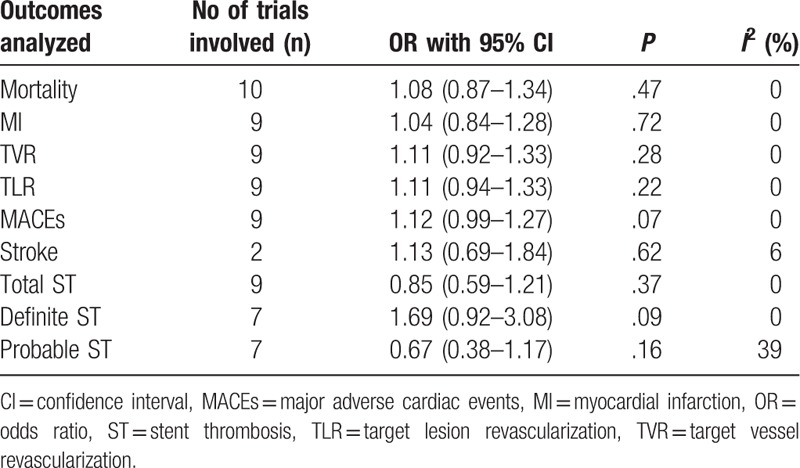
Results of this analysis.

For all of the above analyses, sensitivity analyses yielded consistent results. Except for the fact that when certain trials were excluded and the analysis was carried out, results for MACEs only reached statistical significance, but were not statistically significant. When BIOFLOW II trial was excluded and an analysis was performed, MACEs favored DP-EES and the result reached statistical with OR 1.14, 95% CI 1.00–1.30, *P* = .05. When CENTURY trial was excluded, the result for MACEs again favored DP-EES with OR 1.14, 95% CI 1.00–1.29, *P* = .05. However, exclusion of other trials did not affect the results.

**Figure 7:**
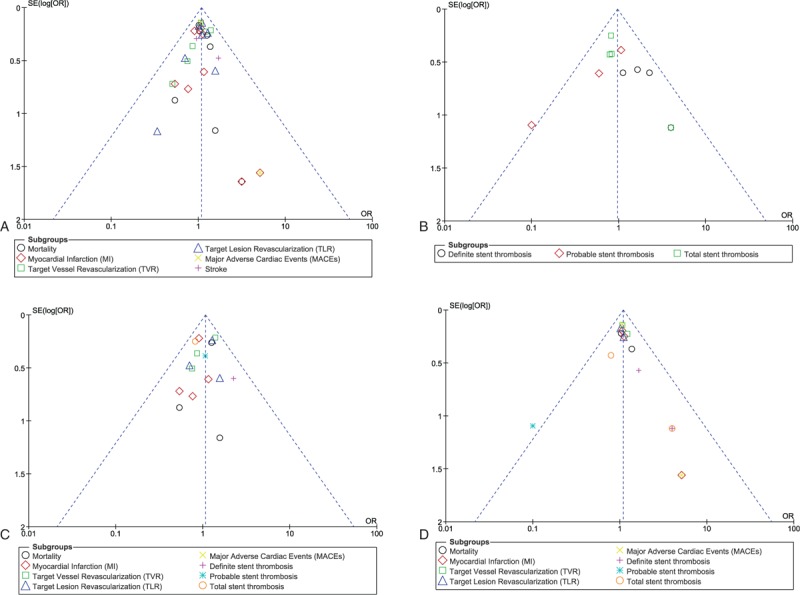
(A–D) Funnel plots representing publication bias.

Based on a visual inspection of the funnel plots obtained, there has been very little evidence of publication bias among the trials that assessed all clinical and cardiovascular endpoints (mortality, MI, TVR, TLR, MACEs, stroke, and ST). The funnel plots showing publication bias have been illustrated in Figs. 7A–D.

## 4 Discussion

This analysis aimed to compare BP-DES with DP-EES in patients with coronary artery diseases (CADs). The results of this analysis showed that BP-DES were noninferior to DP-EES in terms of adverse cardiovascular events. BP-DES and DP-EES were associated with similar rates of mortality, MI, MACEs, stroke, and repeated revascularization during a mean follow-up ranging from 6 months to 3 years. Total ST was also similarly manifested between these biodegradable and nonbiodegradable intracoronary stents. However, even if definite ST was higher in the BP-DES group, the result was not statistically significant. Moreover, even if probable ST was insignificantly higher in the DP-EES group, a moderate heterogeneity was observed in this particular subgroup. Even when BP-SES and BP-BES were separately compared with DP-EES, no significant difference was observed in the results.

Similar to the results of this current analysis, another meta-analysis comparing BP-DES with DP-EES and involving only 4 trials with a total number of 8282 patients showed that BP-DES were noninferior to DP-EES in terms of MACEs and ST.^[17]^ Moreover, the observational study including a total number of 707 consecutive patients with ST segment elevated MI also showed BP-DES to report similar adverse outcomes compared to DP-EES during a follow-up period of 2 years.^[18]^ Another study involving data from the Korea Acute Myocardial Infarction Registry (KAMIR) including a total number of 3359 patients with acute MI showed BP-SES to be noninferior to second-generation DP-DES during a follow-up of 2 years.^[19]^ Recently, even Pandya et al^[20]^ showed no significant differences between BP-DES and second generation DP-DES. However, their meta-analysis not only included DP-EES, but also included DP-ZES and the patients were followed up for a mean time period of 16 months only.

In addition, the meta-analysis of randomized trials comparing the effectiveness and safety between BP-DES and DP-DES showed no significant reduction in MACEs with the use of BP-DES.^[21]^ However, a significantly lower risk of late ST was observed in the BP-DES group when compared to DP-DES. Note that among 8 trials which were included, 3 trials involved DP-EES. Also, the study comparing absorbable polymer sirolimus-eluting stents (MiStent) to the DP-EES using patients from the DESSOLVE I/II and ISAR TEST 4 studies showed the former to be associated with reduced clinically indicated TLR, without any change in ST.^[22]^

Nevertheless, it should not be ignored that a short duration (≤6 months) of dual antiplatelet therapy might be sufficient with EES as shown in the recently published meta-analysis,^[23]^ whereby this short treatment duration was considered reasonable, with a low percentage of major bleeding, similar death rate as well as similar ST.

This current meta-analysis showed results which were completely different from previously published network meta-analyses comparing BP-DES with DP-DES including DP-EES. These network meta-analyses showed DP-EES to be associated with better adverse outcomes compared to BP-DES.^[1–3]^ However, results from this current analysis involved data directly obtained from randomized trials and reported a very low risk of bias among several subgroups analyzing the adverse cardiovascular outcomes. Results of this analysis which were different from those network meta-analyses might have been because of the fact that network meta-analyses which are often referred to as mixed treatment comparison meta-analysis (MTC meta-analysis) are considered as extensions that allow direct and indirect comparisons in combinations, which, according to the recommendations from the Cochrane Collaboration, are not considered as randomized, but are considered as “observational findings across trials,” and may therefore suffer the biases reported among observational studies, for example owing to confounding, even if they included high-quality randomized trials.^[5]^

## 5 Limitations

Similar to other studies, this analysis also has limitations. First of all, owing to the limited number of patients, this analysis may not provide excellent results. Second, study Separham which reported cardiac mortality has been assumed to be all-cause mortality and included in the analysis. This might have a mild effect on the results of this current analysis. Moreover, the BP-DES group involved patients treated with different kinds of stents combined together (BP-SES, BP-EES, BP-BES). This could also be a limitation in this analysis which was partly solved when BP-SES and BP-BES were separately compared with DP-EES. Only 2 trials reported stroke. Using only 2 trials to analyze this specific subgroup might also be a limitation in this meta-analysis. Another limitation could be the different follow up periods reported and the duration of anti-platelets which was different in several trials. However, in most of the trials, the follow-up period as well as the duration of anti-platelet treatment was restricted to 1 year.

## 6 Conclusion

Between 6 months and 3 years, BP-DES were similar in terms of cardiovascular outcomes compared to DP-EES. However, further long-term follow-up research is recommended. To be more precise, mortality, MACEs, stroke, and repeated revascularization were not significantly different between biodegradable DES and nonbiodegradable EES. Total ST was also not significantly different between these 2 types of stents. However, even if definite ST insignificantly favored DP-EES, further studies with longer follow-up periods should be recommended to completely solve this issue.

## Acknowledgments

This research was supported by Youth Science Foundation of Guangxi Medical University (No. GXMUYSF201308), Scientific Project of Guangxi Higher Education (No. KY2015ZD028), and National Natural Science Foundation of China (No. 81560046).
